# Ichthyosis: case report in a Colombian man with genetic alterations in *ABCA12* and *HRNR* genes

**DOI:** 10.1186/s12920-021-00987-y

**Published:** 2021-05-26

**Authors:** Ruben D. Arias-Pérez, Salomón Gallego-Quintero, Natalia A. Taborda, Jorge E. Restrepo, Renato Zambrano-Cruz, William Tamayo-Agudelo, Patricia Bermúdez, Constanza Duque, Ismael Arroyave, Johanna A. Tejada-Moreno, Andrés Villegas-Lanau, Alejandro Mejía-García, Wildeman Zapata, Juan C. Hernandez, Gina Cuartas-Montoya

**Affiliations:** 1 Grupo de Investigaciones Biomédicas Uniremington, Programa de Medicina, Facultad de Ciencias de La Salud, Corporación Universitaria Remington, Medellín, Colombia; 2 Grupo OBSERVATOS, Facultad de Educación Y Ciencias Sociales, Tecnológico de Antioquia –Institución Universitaria, Medellín, Colombia; 3 Grupo Neurociencia Y Cognición, Facultad de Psicología, Universidad Cooperativa de Colombia, Medellín, Colombia; 4 Grupo GIOM, Facultad de Odontología, Universidad Cooperativa de Colombia, Medellín, Colombia; 5 Grupo de Genética Molecular (GENMOL), Facultad de Ciencias Exactas y Naturales (FCEN), Universidad de Antioquia UdeA, Medellín, Colombia; 6 Infettare, Facultad de Medicina, Universidad Cooperativa de Colombia, Medellín, Colombia

**Keywords:** Harlequin ichthyosis, Congenital ichthyosis, Ichthyosis, Skin disease, Case report

## Abstract

**Background:**

Ichthyosis is a heterogeneous group of diseases caused by genetic disorders related to skin formation. They are characterized by generalized dry skin, scaling, hyperkeratosis and frequently associated with erythroderma. Among its different types, harlequin ichthyosis (HI) stands out due to its severity. HI is caused by mutations in the *ABCA12* gene, which encodes essential proteins in epidermal lipid transport, and it helps maintain the homeostasis of the stratum corneum of the epidermis. However, due to the wide spectrum of genetic alterations that can cause ichthyosis, holistic medical care, and genetic studies are required to improve the diagnosis and outcomes of these diseases.

**Case presentation:**

Here, we presented the case of a 19 years old male patient who was a premature infant and exhibited clinical features consistent with HI, including bright yellow hyperkeratotic plates with erythematous fissures that covered his entire body like a collodion baby. Currently, he exhibited erythroderma, photosensitivity, ectropion, auricular pavilion alterations, and musculoskeletal disorders, such as equinovarus feet, fingers, hands, and hypoplastic feet with contractures in flexion and marked difficulty in fine motor skills. In addition, he presented dyschromatopsia, Achilles reflex hyporeflexia, slight speech, dental alteration and deficient cognitive performance. After the genetic sequencing, variants were found in *ABCA12* and *HRNR* which are related to several skin diseases, including ichthyosis.

**Conclusions:**

Although in clinical practice, ichthyosis is a common entity, a severe type of ichthyosis is presented, highlighting the importance of appropriate genetic diagnosis, given the broad spectrum of genetic alterations with similar phenotypic and clinical characteristics. These pathologies must be known to guarantee initial support measures to prevent complications and offer multidisciplinary management to those patients.

**Supplementary Information:**

The online version contains supplementary material available at 10.1186/s12920-021-00987-y.

## Background

Hereditary ichthyoses (OMIM: Ichthyosis, congenital, autosomal recessive 4A 601277) are a group of keratinization disorders. The term ichthyosis is derived from the Greek word ichthys, which means fish because people with these diseases are characterized by having dry, scaly and hyperkeratotic skin [1]. The last classification differentiates two major types of ichthyosis: the non-syndromic types, which are manifested exclusively in the skin and the syndromic types, which affect the skin and other organs [2]. Within the non-syndromic types, four subgroups are distinguished: common ichthyoses, autosomal recessive congenital ichthyoses (ARCI), keratinophatic ichthyoses and other forms of ichthyosis, which are less common. In the subgroup of common ichthyoses are ichthyosis vulgaris and recessive X‐linked ichthyosis (RXLI), and usually have a delayed onset. In the subgroup ARCI; lamellar ichthyosis, congenital ichthyosiform erythroderma, and harlequin ichthyosis (HI, OMIM: Ichthyosis, congenital, autosomal recessive 4B 242500) are the most important, see Table 1 [3–6].

**Table 1:** Subtypes of inherited ichthyoses: non-syndromic forms

Phenotypes	Associated genes
Common ichthyosis	
Ichthyosis vulgaris	*FLG, HRNR*
Recessive X-linked ichthyosis	*STS, VCX3A*
Autosomal recessive congenital ichthyosis	
Major types	
Harlequin ichthyosis	*ABCA12*
Lamellar ichthyosis	*ABCA12, ALOXE3, ALOX12B, CERS3, CYP4F22, NIPAL4/ICHTHYIN, PNPLA1, TGM1*
Congenital ichthyosiform erythroderma	*ABCA12, ALOXE3, ALOX12B, CERS3, CYP4F22, LIPN, NIPAL4/ICHTHYIN, PNPLA1, TGM1*
Minor types	
Self-healing collodion baby	*ALOXE3, ALOX12B, TGM1*
Acral self-healing collodion baby	*TGM1*
Bathing suit ichthyosis	*TGM1*

HI is the most severe and aggressive phenotype of ARCI and it is a rare and commonly fatal skin disorder. Approximately 200 cases of HI have been reported in the medical literature; it is estimated that the incidence is around 1 case per 500,000 births and its distribution by sex seems to be the same between males and females [6–8]. HI is caused by mutations in the *ABCA12* gene (ATP-binding cassette subfamily A, member 12), located on the long arm of chromosome 2 (2q35). The *ABCA12* gene codes for a protein of the family of cholesterol transport proteins ATP-dependent, proteins of this family and its processing enzymes are involved in epidermal lipid transport, which is essential to maintain the stratum corneum skin homeostasis [3, 9–11].

The *ABCA12* gene has been associated with important functions in the differentiation of keratinocytes and epidermal morphogenesis, which is why the clinical features are so serious when there is a great alteration in its function [1, 12]. The severity of mutations of the *ABCA12* gene is related to the clinical phenotype; other less serious pathologies such as lamellar ichthyosis and congenital ichthyosiform erythroderma are associated with partial defects in the function of the *ABCA12* gene, in contrast, mutations that produce complete loss of this gene function generate HI [3, 6, 9]. Newborns affected with HI are clinically characterized by extensive hyperkeratotic plates, bright, white or yellow color, that covers the entire body; this is known as collodion membrane and these patients as collodion babies [13]. These plates usually configure patterns in the shape of a diamond and are surrounded by erythematous fissures, which resemble the harlequin costume, a classic character of the Italian comedy of the Middle Ages, hence the name of the pathology [13]. In addition, facial anomalies such as: (1) bilateral ectropion (complete eversion of the eyelids), which generates the risk of corneal ulceration due to dry eyes; (2) eclabium (eversion of the lips) that makes the mouth constantly stills open, making it difficult to feed the newborn and, in some cases, requiring tube feeding; (3) malformations of the auricular pavilion; (4) nasal hypoplasia and absence of eyelashes and eyebrows [8, 13].

Historically, a newborn affected with HI frequently died within a few weeks after birth due to feeding problems, skin infections, electrolyte imbalance, and respiratory failure reaching mortalities around 50% [3]. In addition, a multicenter study reported that newborns’ deaths occurred mainly during the first three months of life because of respiratory failure and sepsis in 75% of cases and reported an overall survival rate of 56% (25 patients) [7]. However, in Japan it was reported 16 cases from 2005 to 2010 with 81.3% (13 patients) survival [14]. There is no cure for this condition, and only supportive treatment can be given to prolong life [9].

On the other hand, neurological and neuropsychological alterations have been reported in some congenital ichthyoses, particularly those known as Neuro-ichthyotic Syndromes [15]. Due to the urgency of guaranteeing physical health in severe ichthyosis, there is not enough information about the mental health problems that may be associated with a diminished quality of life, such as emotional disorders, certain personality traits and neuropsychological dysfunctions that could affect family and social functioning [16]. To our knowledge, this is the first report of neurological, neuropsychological, psychological, dental, physical, and genetic aspects associated with atypical and severe ichthyosis.

## Case presentation

We presented the case of a 19 years old male from Medellin-Colombia, who was born premature (32 weeks of gestation) and showed clinical features consistent with HI, including bright yellow hyperkeratotic plates that covered his entire body. The clinical history evidenced the death of a brother of one month because of a pulmonary malformation without any family history of congenital skin pathologies. However, there is a family history of neurodegenerative diseases. There is no family history of psychological disorders, epilepsy, mental retardation, learning disabilities, Down syndrome, psychomotor development disorder, or attention deficit disorder.

During the first months of his life, he received a multidisciplinary treatment with a poor prognosis. Remained hospitalized during the first month of life, with improved hyperkeratosis, but he remained with hypersensitivity to touch, photosensitivity, and fissures in the palms and soles, developing generalized erythema with persistent peeling. At the age of 5 months, a biopsy was performed, which reported changes suggestive of congenital ichthyosiform erythroderma diagnosis, but genetic studies were not done despite the physician’s recommendations.

**Fig. 1:**
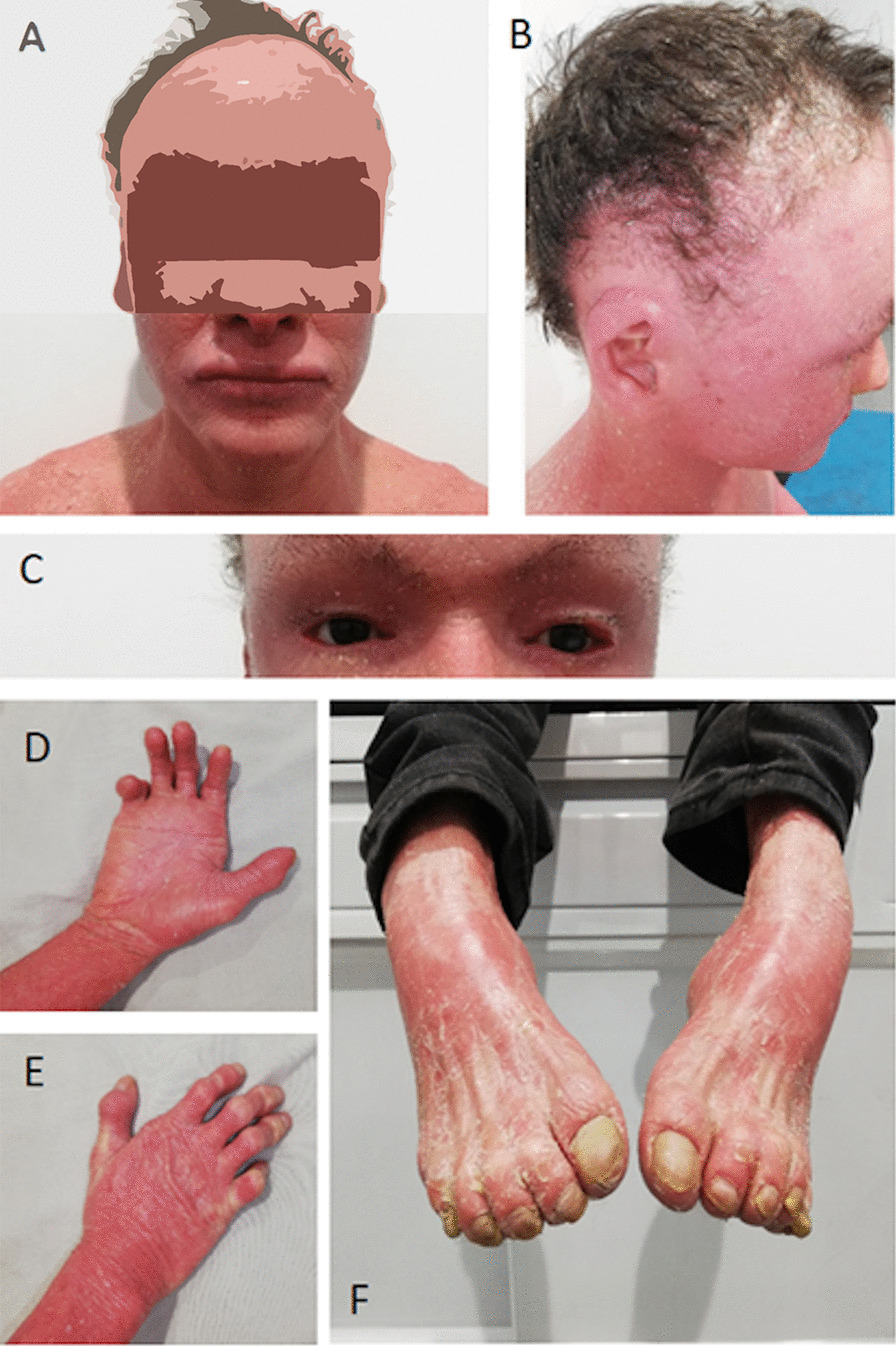
Patient physical characteristics. **a** Generalized erythema with scaling, a marked presence of periorificial wrinkles. **b** Side view of the head where scaly areas on the scalp with ophiasis pattern of alopecia. **c** Poor eyebrow hair and the bilateral ectropion that does not allow closing his eyes completely. **d** Palm view of the right hand, showing the hypoplasia and contractures of the hands with intense scaling. There is an easy development of fissures in hand folds. **e** Back view of the right hand, with a notable deformity in distal bones of the hand with visible microcirculation disturbances due to the contractures in the joints and the skin pressure. **f** Equinovarus feet, erythema, scaling, and onychorrhexis, are observed

Throughout life, the patient presented a short size and low weight (height 1.49 m, weight 41 kg, body mass index 18.5, head circumference 54 cm). He showed signs and symptoms characteristics of HI, such as marked erythema and scaling in all body, frequent scaling of the scalp with scarring alopecia areas, the auricular pavilions had the helix and antihelix fused with the head, corneal opacity in the left eye, atrophy of the optic disc in the right eye and bilateral ectropion and sparse eyebrows on both sides, see Fig. 1a–c. In addition, the patient exhibited a mesosystolic heart murmur in aortic and pulmonary foci of 2/6 grade. Finally, important musculoskeletal findings included the presence of hypoplasia and contractures of the hands and feet; hypoplastic fingers deformed in flexion and atrophy of the hand muscles; equinovarus feet, with hypoplastic fingers feet with flexion contracture, see Fig. 1d–f.

The patient received treatment with oral retinoids (Acitretin) in his childhood, but approximately from 10 years of age, his only treatment for the skin is the use of petroleum jelly (Vaseline) throughout his body after bathing, in addition, he does not use any type of antisolar or moisturizer on the skin. He is not currently receiving any treatment for ectropion, such as artificial tears or other eye lubricants.

### Neuropsychological evaluation

The main findings of the neurological examination were: When applying color vision tests (Farnsworth D-15 color test and Ishihara test), red and green vision disorder was evident, accused limitation for the mobility of fingers and toes, mild gait difficulty, left and right Achilles reflex hyporeflexia, serious alterations in fine motor skills (finger opposition movements, rapid alternating movements, coordination) of left and right superior and inferior limb and slight speech alteration. Regarding behavior, moderate levels of aggression, irritability, isolation, and apathy were identified. A complimentary assessment by a clinical psychologist also reported an intermediate level of apathy and a high level of inability to concentrate.

**Fig. 2:**
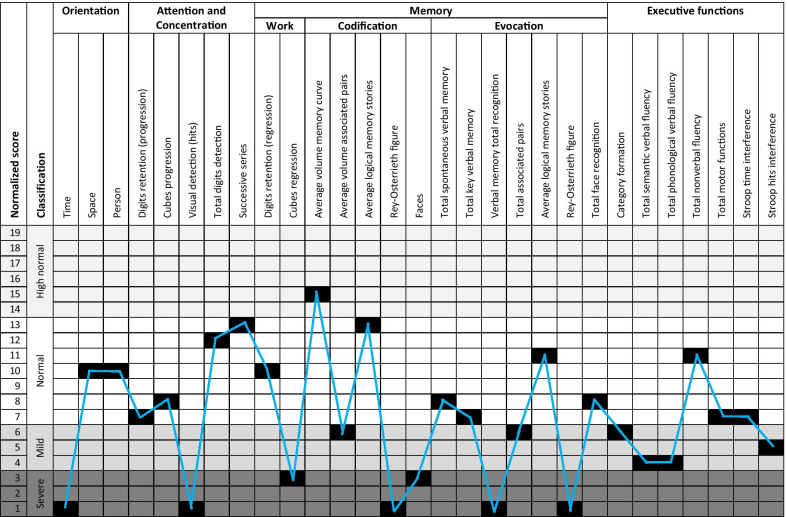
Neuropsi test results. The 26 tasks included assessing each neuropsychological function (attention, memory, and executive functions). Most of the results showed mild and severe alterations. Some others were at a low average level

Executive functioning, memory, and attention were assessed using Neuropsi (Fig. 2), a standardized neuropsychological test [17]. The standardized score for attention and executive functions was 57, for memory it was 49, and for attention and memory, it was 45. These three neuropsychological functions, according to age and schooling, had severe alterations when compared with the general population. Anxiety and depression were assessed using the Beck Anxiety Inventory [18] and Beck Depression Inventory [19]. Anxiety, with a score of 9, was at the intermediate level and depression, with a score of 23, at a moderate level. The level of stress, assessed through the Stress Assessment Score [19], was very low (score 34). Regarding personality [20], the NEO FFI test was used, showing high levels of neuroticism (pth 95) and openness to experience (pth 80), low levels of extraversion (pth 1) and agreeableness, (pth 2) and normal level of consciousness (pth 50). Additionally, to evaluate the association of the neuropsychological state of this patient with physiological functions; the cortisol, serotonin, and tryptophan were determined in serum samples in the reference laboratory, Prolab-Synlab from Medellin-Colombia. The values obtained were into the reference range for each parameter, 9 µg/dL, 106.1 µg/L, and 49.7 µmol/L; respectively.

### Craniofacial development

**Fig. 3:**
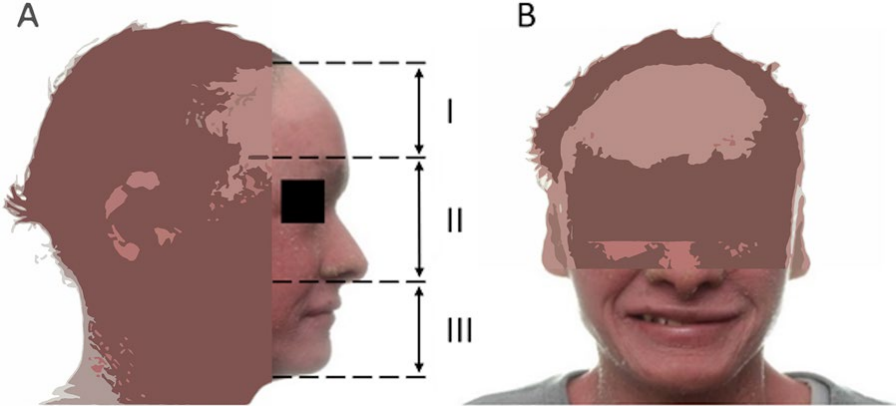
Extraoral photos. **a** Upper facial third from the implantation of the hair to the supraciliary line (80 mm); middle third from the supraciliary line to the base of the nose (75 mm); and lower third from the base of the nose to the lower part of the jaw (50 mm). **b** Mild smile due to lack of elasticity in the skin that limits muscle function

The patient has a brachycephalic cranial type which means that the anteroposterior cranial diameter is shorter than the transverse diameter and presents an euryprosopic facial type (transverse and short wide face). Almost there is a discrepancy and disharmony between the face thirds, finding an increased upper third related to high hair implantation. The analysis of the smile (generated by flexing 17 muscles located around the mouth and eyes), was unable to determine the style of the smile due to lack of elasticity in the skin that limits muscles function; however, a low smile is found according to the position of the upper lip (Fig. 3) [21].

The functional analysis shows a mature swallowing, temporary chewing that indicates little activity of the masseter muscles, probably related to the lack of elasticity of the skin; he does not refer pain in any of the four muscles of mastication at the time of closure and oral opening. It has a maximum diminished mouth opening of 35 mm without pain reported being the normal range of 40 to 50 mm.

**Fig. 4:**
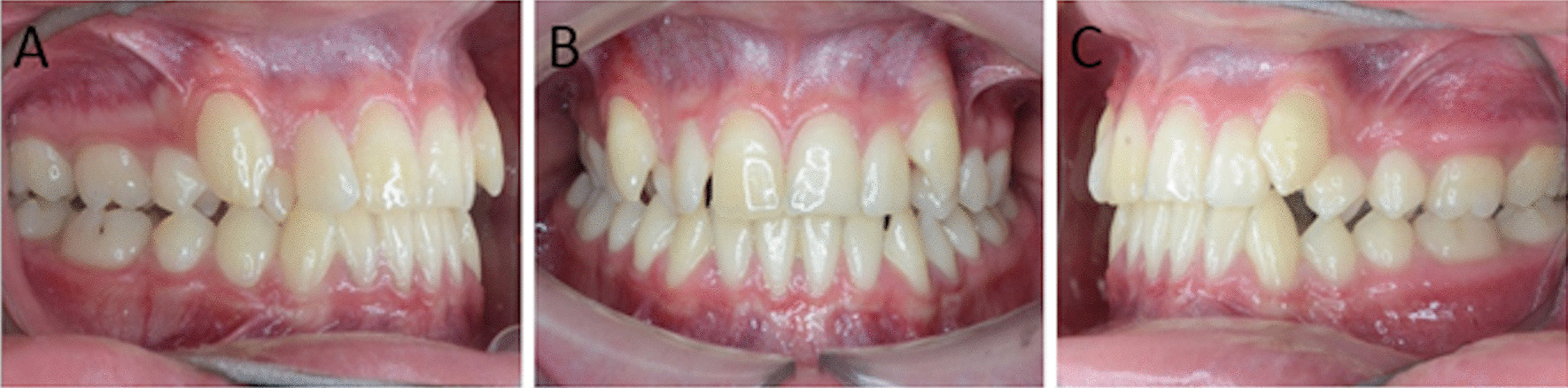
Intraoral photos. Different views of the occlusal type of teeth. **a** Lateral right. **b** Frontal. **c** Lateral left

At the dental level, a permanent dentition type, congenital absence of 1.8.2.8, no dental mobility, or dental anomalies of shape, size and color, were observed. Dental anomalies of position in tooth 1.2 (distoangulated) 1.4 (distal rotation), 1.3 and 2.3 ectopically erupted by perimeter of the diminished arch since the average is 72 mm, were also observed (Fig. 4).

Regarding the jaw sagittal relation analysis among dental arches, the patient presented an Angle’s class II molar relation at 2 mm right and an Angle’s class 1 at 1 mm left. A vertical overbite of 25% and a 1 mm overjet, right and left 2 mm spee curve (an imaginary line which goes from the lower canine distal, passes through the vestibular cusps to the last molar present in the mouth), were observed, indicating immediate and effective anterior guide function without posterior sectors interference possibility, upper midline coincides with the facial one, and a lower midline deviated 2 mm left. Moreover, an oval upper and lower arch shape is observed, and in quadrant 1 and 2 a severe dental crowding [22].

**Fig. 5:**
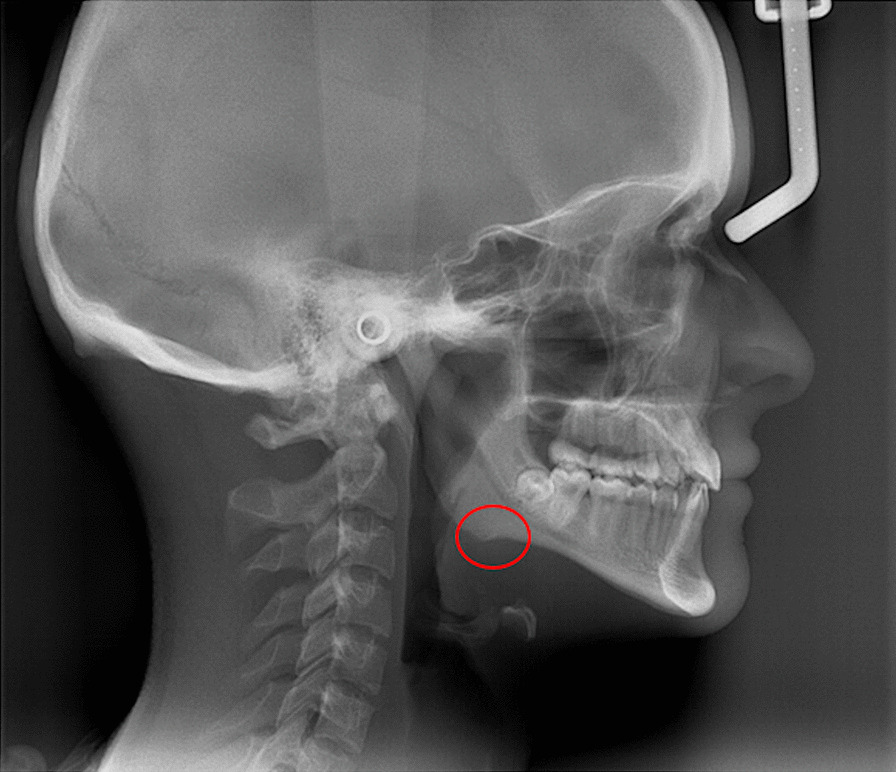
Lateral skull x-ray. The antegonial recess which is related to the pulsatile activity of the facial artery, is observed [23]

A class 1 skeletal relationship is found in the cephalometric analysis; it has a suitable maxillo-mandibular sagittal position; however, the jaw is smaller when compared to the maxillary. In the cephalometric analyses, all vertical dimensions are very low, indicating a significant vertical growth deficiency. From a sagittal view, the very marked antegonial (facial) recess in the mandibular base related to the pulsatile activity of the facial artery, generates an abnormal shape of the lower edge of the same (Fig. 5) [23].

### Genetic analysis

In the exome analysis using Next Generation Sequencing (NGS), we found variants in *ABCA12* and *HRNR* that can be related to the clinical findings observed (Additional file 1).

## Discussion and conclusion

There are mutations of several genes involved in the outcome of congenital ichthyoses, specially ARCI, for that it is necessary to identify the mutation in the patient’s genome to provide a better treatment [24]. To confirm the diagnosis and gene damage in patients with hereditary ichthyoses, DNA analysis has been used for more than 30 years by the Sanger sequencing method, which has represented challenges due to its high cost and the time necessary for developing the test [25]. Currently, other molecular diagnostic methods have been developed with reliable results, without invasive procedures such as skin biopsy, which may have more repercussions, especially in such patients [26]. Prenatal diagnosis is also possible, the identification of the gene mutation with DNA analysis by chorionic villus or amniotic fluid cell sampling at earlier stages of pregnancy or diagnostic using 3D/4D ultrasound since these methods can be observed signs suggestive of hereditary ichthyoses [27].

Respiratory failure is the main cause of death in newborns affected by HI, attributed to rigidly adherent scales on the thorax, or maybe defective alveolar surfactant secretion due to *ABCA12* defects [27]. However, with the increased availability of neonatal intensive care units and the early administration of retinoid therapy, a marked reduction of mortality was achieved, as 80% of cases that receive timely and adequate treatment survived [14]. There are no curative treatments for HI, but systemic retinoid has been used with good results, especially acitretin, because its shorter half‐life offers a safety profile. In neonates with HI, early induction of systemic retinoid promotes accelerated shedding of the hyperkeratotic plates, and constant use decreases scaling and improves ectropion and eclabium [27].

Children who survive the neonatal period have an average life expectancy and tend to develop intense erythroderma, like severe congenital ichthyosiform erythroderma with ocular complications related to persistent ectropion, limitations in growth, and a limitation of fine motor skills, and they present problems in social relationships, which affect the quality of life in these patients [4, 11, 14], as observed in this patient.

Regarding the genetic analysis, we look for variants in *ABCA12* gene because this gene is the most consistently associated with the phenotype. Although no pathogenetic mutations were identified in *ABCA12*, a synonymous variant considered potentially damaging, potentially pathogenic, and potential alteration of splicing was found. Previous studies have shown that this phenotype can be caused by synonymous variants [1, 28, 29]. As the case of the homozygous synonymous mutation in exon 24 (c.3456G>A; p.S1152S) in *ABCA12*, reported in a consanguineous family of Arab Muslim origin with several members displaying a severe form of congenital ichthyosiform erythroderma, which was found to lead to the formation of a novel splicing acceptor [28]. These mutations can create de novo splicing sites, leading to premature protein translation and altering its normal function, which may explain the phenotypic expression. The variant we found has enough in silico support about splicing alteration to be considered a candidate variant; however, expression studies are needed to confirm this hypothesis.

As the family members are unaffected, and no candidate variant in the homozygous state was identified, we looked for compound heterozygotes. We found two variants in the *VCX3A* gene. This gene belongs to the VCX/Y gene family, which has multiple members on both X and Y chromosomes, and all are expressed exclusively in male germ cells. There are reports of microdeletions in *VCX3A* in families with *X-linked ichthyosis*; however, those microdeletions also interrupt a close gene called STS, which is responsible for the phenotype [27]. We also found 10 variants in the *HRNR* gene, two of them were frameshift variants, classified as VUS. Both variants generate premature stop codons, and the resulting protein would lack the last two repeats of the protein. The *HRNR* gene is associated with *Ichthyosis vulgaris* and *atopic dermatitis*; the protein has been purified from stratum corneum in healthy skin [27]. In vitro models of *Ichthyosis vulgaris* showed that hornerin (*HRNR*) expression is decreased, suggesting a link between the causal gene *FLG* and *HRNR*. FLG and *HRNR* are fusedS100 proteins and they are part of the epidermal differentiation complex and components of the cornified envelope (stratum corneum) HRNR is thought to be a causative gene because it is strongly reduced in *Ichthyosis vulgaris* compared with healthy skin [30].

Considering that de novo mutations could explain that the patient is the only individual affected within the family, we also looked for variants in heterozygous state. Eighteen variants were found, including one located in the *KRT6B* gene (protein encoded by this gene is a type 2 cytokeratin involved in the differentiation of simple and stratified epithelial tissues). This gene is related to *Pachyonychia congenita*, a disease that causes nail dystrophy, and the fingernails and toenails become thick and abnormally shaped. Although the gene is related to alterations in keratin, the variant found is classified as likely benign. The variant found in the *TGM3* gene is classified as likely benign. The gene product is a transglutaminase, and it is involved in the later stages of cell envelope formation in the epidermis and hair follicle. This gene is associated with *Uncombable hair syndrome*. Other TGM genes are related to ichthyosis, though the *TGM3* gene has not been associated with this disease [31]. A variant in the *COL7A1* classified as uncertain significance, is believed to alter splicing, but the exact effect is unclear. The gene product encodes for the alpha chain of type VII collagen and it is associated with *Epidermolysis Bullosa Pruriginosa*. This disease is characterized by hypertrophic plaques in a linear configuration, in the lower extremities and the lesions are pruritic. However, there are reports of exome data that have found that variants in the *COL7A1* could segregate along with variants in the *FLG* gene, but only the last one is responsible for Ichthyosis [32]. Another VUS was found in *DYSF* gene, and it is classified as likely pathogenic. However, this protein encodes a skeletal muscle protein found associated with the sarcolemma related to *muscular dystrophy*. There are reports of patients with *ichthyosis* and *dysferlinopathy*, but only the last disease is related to *DYSF* gene [33].

In conclusion, although no pathogenetic mutations were identified in *ABCA12* gene, the synonymous variant c.3054C>T, p.G1018G considered as potentially pathogenic can induce a potential alteration of splicing according to bioinformatic analysis. Two other candidate variants are the recessive compound heterozygous variants in the *HRNR* gene since this gene is downregulated in patients with ichthyosis.

As limitations of our study, we only have DNA samples from the index case, which does not allow us to perform a segregation analysis of the candidate variants in the parents or other family members.

On the other hand, some of the sensory and motor alterations detected in the neurological examination may be due to the physical conditions described in the clinical case. For instance, deficits in the perception of colors could be associated with leukocoria or atrophy of the optic disc. In addition, alterations in upper and lower limbs mobility, fine motor skills, and gait would be associated with the presence of hypoplasia and contractures of the hands and feet, atrophy of the muscles of the hand, and hypoplastic fingers.

Similarly, the cranial and facial growth has been altered by the modified characteristics of the skin that have not allowed the development and normal growth of the maxillary skull complex, since the cephalometric and facial measures evaluated, are below standard measurements. Also, in many syndromes with extensive skin lesions, there is a delay in bone age in which the growth retardation becomes more evident with increasing age [34, 35].

However, the neurological findings would not fully explain the results of the neuropsychological assessment. Neither are there any hints in the personal and family history to understand this deficient cognitive performance. His emotional state was not sufficiently altered to affect cognitive performance. Apathy, demotivation, and concentration problems may explain these results. A second neuropsychological assessment would be necessary to verify this hypothesis.

The findings of personality traits are coherent. High levels of neuroticism are associated with depression, introversion, and low agreeableness (which implies low empathy and related social behaviors like cooperation). High levels of neuroticism and low levels of extraversion are characteristic of avoidant and defensive personality styles [36]. These people tend to be unmotivated and insensitive to rewards. High levels of neuroticism may also explain low neuropsychological performance in memory, attention, and executive functioning [37]. These personality traits are also associated with poor quality of life and interpersonal distress [38]. In conclusion, here we report a case of a patient with an initial diagnosis of HI, and after the genetic sequencing, we discovered that the patient presented ichthyosis associated with alteration in the *ABCA12* and *HRNR* genes. The patient presents severe ichthyosis, erythroderma, dysmorphic features, and deficient cognitive performance. The physicians must be informed about the wide spectrum of mutations according to the clinical features of patients to provide the most appropriate diagnostic and therapeutic options, because these patients require a multidisciplinary team for better outcomes. This case illustrates the complexity of interpreting the physical and neurobehavioral phenotype of patients with genetic variants in *ABCA12* and *HRNR* genes.

## Supplementary Information

**Additional file 1.** Genetic evaluation by using Next Generation Sequencing and bioinformatic tools, including FastQC, Burrows-Wheeler Aligner, GATK, SnpEff, wANNOVAR, and Varsome.

## Data Availability

The datasets used and/or analyzed during the current study are available at figshare: https://doi.org/10.6084/m9.figshare.13076309.v1.

## Abbreviations

ABCA12: ATP binding cassette subfamily A member 12
ARCI: Autosomal recessive congenital ichthyosis
COL7A1: Collagen Type VII Alpha 1 Chain
DNA: Deoxyribonucleic acid
DYSF: Dysferlin
HRNR: Hornerin
HI: Harlequin ichthyosis
KRT6B: Keratin 6B
FLG: Filaggrin
NEO FFI: NEO Five-Factor Inventory
NGS: Next Generation Sequencing
RXLI: Recessive X‐linked ichthyosis
STS: Steroid sulfatase
TGM: Transglutaminase
VUS: Variant of uncertain significance
