# Supplementary material for: Ichthyosis: case report in a Colombian man with genetic alterations in ABCA12 and HRNR genes
Source: BMC Med Genomics. 2021 May 26;14:140. doi: 10.1186/s12920-021-00987-y (PMC8157432; doi:10.1186/s12920-021-00987-y)
Supplement: Supplementary file 1 — Additional file 1. Genetic evaluation by using Next Generation Sequencing and bioinformatic tools, including FastQC, Burrows-Wheeler Aligner, GATK, SnpEff, wANNOVAR, and Varsome. [file 12920_2021_987_MOESM1_ESM.docx]

**Genetic Analysis**

**DNA samples and Exome sequencing**

DNA was isolated from peripheral blood following standard extraction protocols (salting out) and stored at -20°C until the time of sequencing. The coding regions of the genome were sequenced by Next Generation Sequencing (NGS) in Macrogen company. The samples were prepared according to an Agilent SureSelect Target Enrichment Kit preparation guide and the libraries were sequenced with Illumina platform sequencer (2 x 101 base pair paired end reads) (1). Finally, the data was processed by the software HCS (HiSeq Control Software) to obtain the raw data. The data product of the sequencing was converted to the format FASTQ using the package Illumina bcl2fastq.

**Bioinformatic analysis**

The quality of reads was evaluated with FastQC v0.11.5 tool of the Babraham Institute (2). Then, reads were mapped against the reference human genome (UCSC hg19) using the Burrows-Wheeler Aligner tool, bwa-0.7.12 (3). Variant calling was performed following the Broad Institute’s Genome Analysis Tool Kit GATK tool (GATK) GATK v3.4.0 Best Practices for Germline SNP & Indel Discovery in Whole Genome and Exome Sequence (4). Duplicate read removal, local sequence realignment and base quality recalibration were performed by Picard-tools-1.130 and GATK v3.4.0. Variants were called using GATK haplotype caller and filtered using the default criteria for variant filtration tool. Variants were annotated with SnpEff tool v4.1g (5).

Additionally, we use wANNOVAR tool of Wang Genomics Lab (6) for the annotation of the variants and Geneyx analysis tool of Gene Cards of the Weizmann Institute of Science (7) to prioritize candidate variants according to the patient's phenotype. Also, we seek the clinical interpretation of the variants using the Varsome (8) of the global human genomic community and wIntervar (9) platforms of the American College of Medical Genetics and Genomics and the Association for Molecular Pathology (10).

The candidate variants were selected considering the following criteria: 1. Quality of the sequences: Depth across samples (DP<30). 2. Gene panel: candidate genes from The Gene Cards Human Data Base 3. Type of variant: Only synonymous variants were considered. 4. Inheritance pattern: Autosomal recessive 5. Allelic frequency (MAF <0.01) in a population database (1000 Genomes, ExAC, ESP6500, gnomAD) 6. Pathogenicity Predictors: variants cataloged as deleterious or possibly deleterious by more than three pathogenicity predictors including SIFT and Polyphem2 and CADD values higher than 14.

**Results**

**Variant identification**

In the exome analysis, a total of 83,058,764 reads were identified corresponding to a total of 12,368,063,578 base pairs. 108963 variants were found, 94,847 SNPs and 14,116 INDELs. Of them 11,404 (27%) correspond to synonymous variants, 10,973 (58%) to missense variants, 33 (2%) to Stop Lost variants, 91 (2%) to Stop Gained, 325 (9%) to frameshift variants, 157 (1%) to Inframe Insertion and 198 (1%) to Inframe Deletion variants. Figure 1. 96.1% are found in dbSNP142 database. The ratio of number of heterozygous variants to number of homozygous variants was 1.3 and the ratio of transition rate of SNVs that pass the quality filters divided by transversion rate of SNVs that pass the quality filters was 2.2.


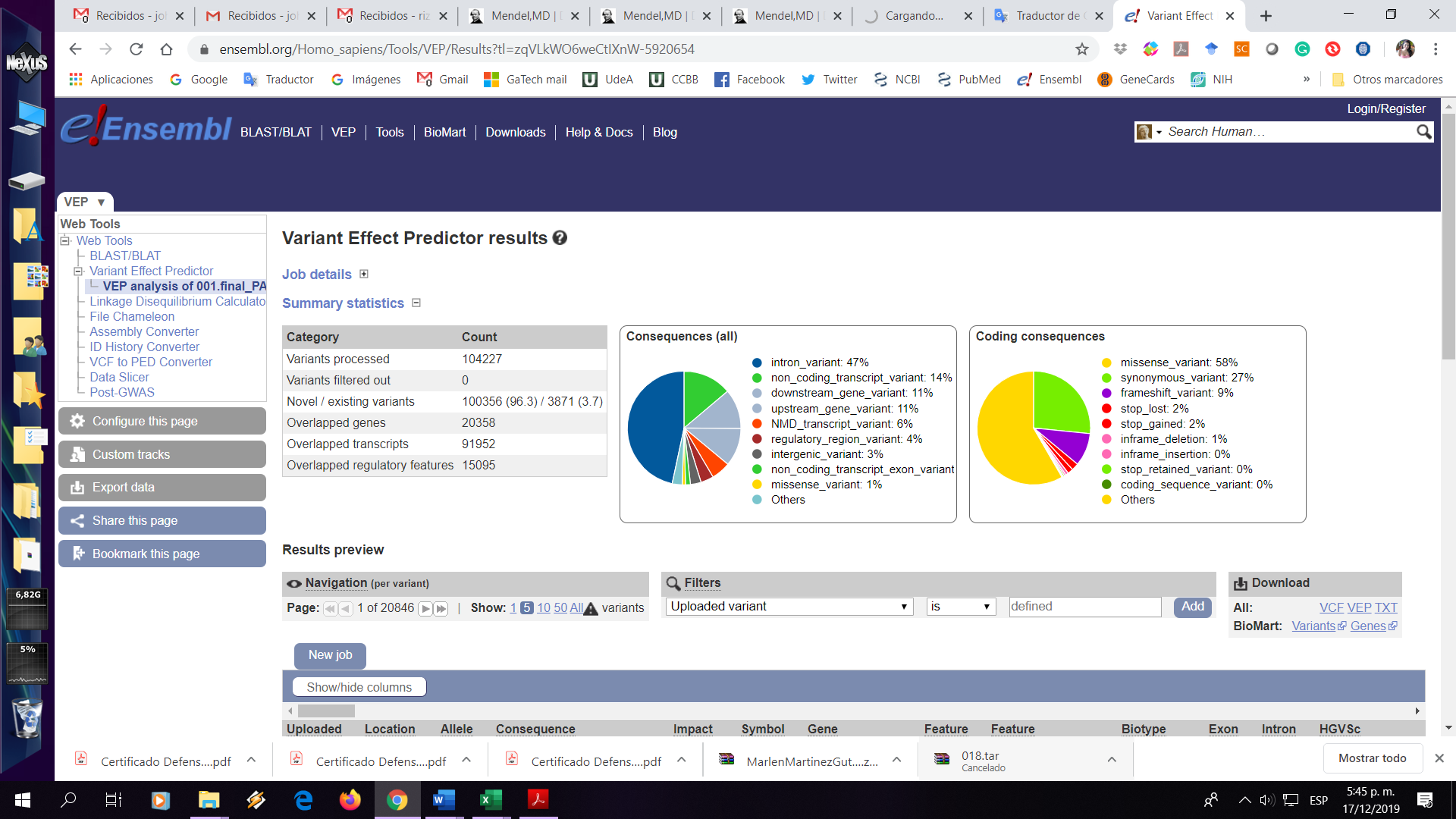


Figure 1. Description of variants identified with the VEP tool in the index case with Ichthyosis.

Considering that the ABCA12 gene is the most consistently associated with the ichthyosis phenotype, we first check for variants in this gene. No pathogenetic mutations were identified in any of the 55 exons of the ABCA12 gene. We found one nonsynonymous variant c.T2329A:p.S777T and two synonymous variants c.C6306T:p.Y2102Y and c.C3054T:p.G1018G, Table1. The nonsynonymous variant c.T2329A:p.S777T is considering as tolerant or benign by the pathogenicity predictors SIFT and Polyphen2 and classified as benign according AMCG criteria. The first synonymous variant c.C6306T:p.Y2102Y is classified as benign by Varsome and wIntervar platforms and the second synonymous variant c.C3054T:p.G1018G is classified as VUS by Varsome platform and as likely benign by wIntervar platform, Table 2.

**Candidate variant in ABCA12 gene**

| **Genetic variant Information** | | | | | | | **Allele Frequency** | | | **Pathogenicity predictors** | | | **GT** |
| --- | --- | --- | --- | --- | --- | --- | --- | --- | --- | --- | --- | --- | --- |
| **Gene** | **Chr** | **Ref** | **Alt** | **Type** | **dbSNP** | **Change** | **1000G** | **ExAC** | **gnomAD** | **SIFT** | **PolyPhen2** | **CADD** |  |
| ABCA12 | Chr2 | G | A | synonymous SNV | rs10498027 | exon43  c.C6306T  p.Y2102Y | 0.35 | 0.4051 | 0.4065 | - | - | - | 1/1 |
| ABCA12 | Chr2 | G | A | synonymous SNV | - | exon22  c.C3054T  p.G1018G | - | - | - | - | - | - | 1/1 |
| ABCA12 | Chr2 | A | T | nonsynonymous SNV | rs7560008 | exon17  c.T2329A  p.S777T | 1. | 0.9994 | 0.9995 | T | B | 0.082 | 1/1 |

Table 1. Description of candidate variant identified in ABCA12 gene with the ANNOVAR tool in the index case with Ichthyosis. **Chr:** Chromosome, Ref: Reference allele, **Alt:** Alternate allele, **dbSNP:** NCBI SNP database, **Change:** nucleic acid/amino acid change, **1000G:** 1000 genomes database, **ExAC:** Exome Aggregation Consortium, **gnomAD:** genome aggregation database, **SIFT:** Sorting Intolerant from Tolerant tool, **PolyPhen2:** Polymorphism Phenotyping v2 tool, **CADD:** Combined Annotation Dependent Depletion tool. **GT:** Genotype. **T:** Tolerant. D: Deleterious. **B:** Benign. **P:** Pathogenic.

**Clinical interpretation of ABCA12 candidate variant**

| **Gene** | **Chr** | **dbSNP** | **Change** | **Clinvar** | **Varsome** | **Intervar** | **ACMG** | **Effect** |
| --- | --- | --- | --- | --- | --- | --- | --- | --- |
| ABCA12 | Chr2 | rs10498027 | Y2102Y | B | BA1, BP4, BP6, BP7 | BA1, BS1, BS2, BP4, BP6, BP7 | B | synonymous SNV |
| ABCA12 | Chr2 | - | G1018G | - | PM2 | PM2, BP4, BP7 | VUS, LB | synonymous SNV |
| ABCA12 | Chr2 | rs7560008 | S777T | B | BA1, BP4, BP6, PP2, PP5 | BA1, BS1, BS2, BP4, BP6 | B | Missense |

Table 2. **Clinical interpretation of the ABCA12 candidate variant identified in the exome analysis in the index case with ichthyosis**. **Gene**: Name of the gene. **Chr:** Chromosome. **dbSNP:** dbSNP database variant Identifier. **Change:** Amino acid change. **ClinVar:** Clinical significance in ClinVar. **Varsome:** Classification of variants according to the Varsome platform. **Intervar:** Classification of the variants according to the Intervar platform. **ACMG:** Classification of the variants according to the American College of Medical Genetics. **B:** Benign, **LB:** Likely benign, **LP:** Likely pathogenic, **P:** Pathogenic, **VUS:** Variant with Uncertain Significance. **Effect:** The effect of the variant in the protein (including splicing effects). **B:** Benign, **LB:** Likely benign, **LP:** Likely pathogenic, **P:** Pathogenic, **VUS:** Variant with Uncertain Significance, **CIOP:** Conflicting interpretations of pathogenicity. **BA1:** Benign Stand-Alone, **BS1-BS4**: Benign Strong, **BP1-BP7**: Benign supporting, **PP1-PP5:** Pathogenic supporting, **PM1-PM6:** Pathogenic moderate, **PS1-PS6:** Pathogenic strong, **PVS1:** Pathogenic very strong.

We use three tools to determine the impact of the synonymous variants located at ABCA12 gene, the first one, TraP, uses the conservation score of the nucleotide, features like the creation of cryptic splice sites, creation, and disruptions of cis-acting binding sites, above all. Taking the recommended cutoff point of 0.459 ss the authors, the variant Chr2:215865554G>A is classified as possibly damaging with a score of 0.534. The SilVA tool uses sequence features like CpG and relative mRNA position, exon splicing enhancer or suppressor, splice site motifs and pre-mRNA folding energy among others, to determine the impact of the synonymous variant. According to this tool, this variant is potentially pathogenic. Finally, the human splicing finder (HSF) predicts the possible effects on splicing, based on splicing donor/acceptor sites, exonic splicing enhancer (ESE) o exonic splicing silencers (ESS). This tool reported that the variant alters de ESE/ESS motifs ratio and it provokes a new donor splice site (activation of a cryptic donor site); HSF concludes that the variant causes “potential alteration of splicing”, Table 3.

**Splicing alteration predictions**

| **Genetic variant Information** | | | **Splicing alteration predictors** | | |
| --- | --- | --- | --- | --- | --- |
| **Gene** | **Chr** | **dbSNP** | **TraP** | **SilVa** | **HSF** |
| **ABCA12** | Chr2 | rs10498027 | Score: 0.031  No pathogenic | Likely benign | Alteration of auxiliary sequences |
| **ABCA12** | Chr2 | - | 0.534  Possibly damaging | Potentially pathogenic | New Donor splice site  Activation of a cryptic Donor site  Potential alteration of splicing |

Table 3. **Clinical interpretation of the ABCA12 candidate variant identified in the exome analysis in the index case with ichthyosis.** **Gene:** Name of the gene. **Chr:** Chromosome. **dbSNP:** dbSNP database variant Identifier. **TraP:** Transcript-inferred Pathogenicity tool. **SilVA:** Silent Variant Analyzer. **HSF:** Human Splicing Finder.

We use wANNOVAR tool for the annotation of the variants and Geneyx analysis tool to prioritize candidate variants according to the patient's phenotype (Ichthyosis). Considering that the patient's parents and siblings are healthy, homozygous variants were initially reviewed assuming an autosomal recessive inheritance pattern and we found 10 recessive homozygous candidate variants, Table 3. Additionally, we seek the clinical interpretation of the variants using the Varsome and wIntervar platforms following the ACMG criteria. No direct association with the phenotype was found according to Geneyx analysis tool, however all the variants seem to have a high effect on the protein, Table 4.

**Recessive homozygous candidate variant**

| **Genetic variant Information** | | | | | | **Allele Frequency** | | | **Evolutionary conservation predictors** | | | **Pathogenicity predictors** | | |
| --- | --- | --- | --- | --- | --- | --- | --- | --- | --- | --- | --- | --- | --- | --- |
| **Gene** | **Chr** | **Ref** | **Alt** | **dbSNP** | **Change** | **1000G** | **ExAC** | **gnomAD** | **Geno**  **Canyon** | **fitCons** | **GERP++** | **SIFT** | **Poly**  **Phen2** | **CADD** |
| USP17L10 | 4 | C | T | rs767409378 | R402* | - | 0.0006 | 0.0003 | 0.000 | 0.554 | -0.674 | - | - | 35 |
| RIC8A | 11 | C | G | rs34925440 | H272Q | 0.006 | 0.0115 | 0.0118 | 1.000 | 0.722 | -5.57 | T | P | 20.8 |
| TAS2R14 | 12 | CAG | C | rs750931419 | L245 | - | 9.904e-05 | 0.0002 | - | - | - | - | - | - |
| ETFBKMT | 12 | G | A | rs138229541 | - | - | - | - | - | - | - | - | - | - |
| PUS7L | 12 | G | A | rs189799390 | R507C | 0.0006 | 8.239e-05 | 4.468e-05 | 1.000 | 0.706 | 5.02 | D | D | 35 |
| RIPK3 | 14 | T | TG | rs531266348 | P492P? | 0.0024 | 0.0025 | 0.0026 | - | - | - | - | - | - |
| MYO1C | 17 | G | C | rs193155058 | - | - | - | - | - | - | - | - | - | - |
| HELZ2 | 20 | G | A | rs139845169 | Q131* | - | 2.836e-05 | 1.291e-05 | 1.000 | 0.660 | 2.4 | - | - | 35 |
| COL18A1 | 21 | C | T | rs372133935 | G516 | - | 8.868e-05 | 3.237e-05 | - | - | - | - | - | - |
| OGT | X | T | C | rs747657088 | I535 | - | 2.403e-05 | 0,01165 | - | - | - | - | - | - |

Table 4. **Description of recessive homozygous candidate variant identified with the ANNOVAR tool in the index case with ichthyosis.** **Gene**: Name of the gene. **Chr:** Chromosome. **Ref:** Reference allele. **Alt:** Alternate allele. **dbSNP:** dbSNP database variant Identifier. **Change:** Amino acid change. **1000G:** 1000 genomes database allele frequency. **ExAC:** Exome Aggregation Consortium database allele frequency. **gnomAD**: genome aggregation database allele frequency. **GenoCanyon:** Conservation scores with GenoCanyon tool (Conserved region=scores~1). **fitCons:** Conservation scores with fitCons tool: (Conserved region= ~1). **GERP++RS:** Conservation scores with GERP++RS tool (Conservation region=scores>4.4). **SIFT:** Pathogenicity prediction with SIFT tool: D=Deleterious, T=Tolerated), **Polyphen2HDIV:** Pathogenicity prediction with PolyPhem2 tool for Mendelian disease variants (D=Damaging, P=Possibly Damaging, B=Benign, U=Unknown). **CADD:** Pathogenicity scores with CADD tool (Deleterious=scores>14).

**Clinical interpretation of recessive homozygous candidate variant**

| **Gene** | **Chr** | **dbSNP** | **Change** | **Clinvar** | **Varsome** | **Intervar** | **ACMG** | **Effect** | **Severity** | **Pheno** |
| --- | --- | --- | --- | --- | --- | --- | --- | --- | --- | --- |
| USP17L10 | **Chr** | rs767409378 | R402* | - | BP4 | PM2 | VUS | Nonsense | High | - |
| RIC8A | 4 | rs34925440 | H272Q | - | BS1, BP4 | BS1, BP4, PM1 | VUS | Missense, Splice site region | High | - |
| TAS2R14 | 11 | rs750931419 | L245 | - | BS1 | - | VUS | frameshift deletion | High | - |
| ETFBKMT | 12 | rs138229541 |  | - | PP3 | - | VUS | Intron, Splice site donor | High | - |
| PUS7L | 12 | rs189799390 | R507C | - | BS1, PP3 | PM1 | VUS | Missense | High | - |
| RIPK3 | 12 | rs531266348 | P492P? | - | BS1 | - | LB | frameshift insertion | High | - |
| MYO1C | 14 | rs193155058 |  | B | BA1, BP4, BP6 | - | B | Intron, Splice site region | High | - |
| HELZ2 | 17 | rs139845169 | Q131* | - | PVS1, PM2, PP3 | - | P | Nonsense | High | - |
| COL18A1 | 20 | rs372133935 | G516 | - | BP4, BP7 | PP3 | LB | Splice site region, Synonymous | High | - |
| OGT | 21 | rs747657088 | I535 | - | BP4, BP7, PM2 | BP4, BP7, PM2 | LB | Splice site region, Synonymous | High | - |

Table 5. **Clinical interpretation of the recessive homozygous candidate variant identified in the exome analysis in the index case with ichthyosis**. **Gene**: Name of the gene. **Chr:** Chromosome. **dbSNP:** dbSNP database variant Identifier. **Change:** Amino acid change. **ClinVar:** Clinical significance in ClinVar. **Varsome:** Classification of variants according to the Varsome platform. **Intervar:** Classification of the variants according to the Intervar platform. **ACMG:** Classification of the variants according to the American College of Medical Genetics. **B:** Benign, **LB:** Likely benign, **LP:** Likely pathogenic, **P:** Pathogenic, **VUS:** Variant with Uncertain Significance, **CIOP:** Conflicting interpretations of pathogenicity. **Effect:** The effect of the variant in the protein (including splicing effects). **Severity:** Severity of the impact on the protein based on the effect. **Pheno:** The score for the association between the gene and the phenotype terms (Ichthyosis) according to GTex platform. **B:** Benign, **LB:** Likely benign, **LP:** Likely pathogenic, **P:** Pathogenic, **VUS:** Variant with Uncertain Significance, **CIOP:** Conflicting interpretations of pathogenicity. **BA1:** Benign Stand-Alone, **BS1-BS4**: Benign Strong, **BP1-BP7**: Benign supporting, **PP1-PP5:** Pathogenic supporting, **PM1-PM6:** Pathogenic moderate, **PS1-PS6:** Pathogenic strong, **PVS1:** Pathogenic very strong.

We also searched for compound homozygous variants and found 23 variants in 6 different genes, Table 5, with a direct relationship with the phenotype according to the Geneyx analysis tool, Table 6. Most of them have a MAF minor 0.01 but only four variants (rs74393938, rs773518265, rs3958533, and rs200979099) are considered as deleterious or possibly deleterious by the pathogenicity predictors. Six variants are classified as benign, nine as likely benign and eight as variant with uncertain significance according ACMG criteria. Four of the variants (rs746911441, rs776354084, rs3958533 and rs372013175) seem to have a high effect on protein, for the others the effect is considered as medium.

**Recessive compound heterozygous candidate variant**

| **Genetic variant Information** | | | | | | | **Allele Frequency** | | | | **Evolutionary conservation predictors** | | | | **Pathogenicity predictors** | | | |
| --- | --- | --- | --- | --- | --- | --- | --- | --- | --- | --- | --- | --- | --- | --- | --- | --- | --- | --- |
| **Gene** | **Chr** | **Ref** | **Alt** | **dbSNP** | **Change** | **1000G** | | **ExAC** | **gnomAD** | **Geno**  **Canyon** | | **fitCons** | **GERP++** | **SIFT** | | **Poly**  **Phen2** | **CADD** |  |
| VCX3A | X | C | T | rs74393938 | V186M | - | | 0.0109 | 0.0001 | 0.000 | | - | 0.463 | D | | D | 7.585 |  |
| VCX3A | X | * | - | - | V160_E179del | - | | 0.0002 | 0.0010 |  | |  |  |  | |  |  |  |
| HRNR | 1 | T | A | rs773518265 | Q2469L | - | | 0.5 | 0.3203 | 0.000 | | 0.554 | 1.18 | D | | P | 8.576 |  |
| HRNR | 1 | T | A | rs12116427 | T2270S | - | | 0.1069 | 0.0252 | 0.000 | | 0.554 | -2.27 | T | | B | 0.007 |  |
| HRNR | 1 | GATCC | G | rs746911441 | GS2267 | - | | 0.0038 | 0.0006 | - | | - | - | - | | - | - |  |
| HRNR | 1 | C | CAT  GT | rs776354084 | Q2265QH? | - | | 0.0045 | 0.0005 | - | | - | - | - | | - | - |  |
| HRNR | 1 | T | C | rs747830959 | D2264G | - | | 0.0021 | 0.0002 | 0.000 | | 0.554 | 0.55 | T | | B | 1.479 |  |
| HRNR | 1 | A | C | rs769619529 | F2263C | - | | 0.0011 | 7.163e-05 | 1.000 | | 0.554 | -5.38 | T | | B | 0.001 |  |
| HRNR | 1 | G | C | rs1266501645 | T2262S | - | | - | - | - | | - | - | - | | - | - |  |
| HRNR | 1 | G | A | rs139799138 | R2167C |  | | 0.0657 | 0.0144 | 0.039 | | 0.554 | -5.36 | T | | B | 4.599 |  |
| HRNR | 1 | C | G | rs186602563 | S860T | 0.001 | | 0.0008 | 0.0010 | 0.000 | | 0.487 | 2.27 | T | | P | 2.134 |  |
| HRNR | 1 | T | C | rs76694305 | T616A | 0.0002 | | 1.647e-05 | 0.0039 | 0.032 | | 0.554 | -7.45 | T | | B | 0.002 |  |
| HS6ST1 | 2 | G | T | rs3958533 | R249S | - | | 0.3522 | 0.1439 | 1.000 | | 0.610 | 5.06 | D | | D | 32 |  |
| HS6ST1 | 2 | A | C | rs199993343 | V114G | - | | 0.2274 | 0.1165 | 1.000 | | 0.767 | 3.69 | T | | D | 22.7 |  |
| HS6ST1 | 2 | G | T | rs200979099 | D87E | - | | 0.4625 | 0.4245 | 1.000 | | 0.767 | 2.78 | D | | D | 27.5 |  |
| DSG4 | 18 | C | T | rs36040686 | S79L | 0.0044 | | 0.0042 | 0.0044 | 0.004 | | 0.487 | -1.72 | T | | B | 9.626 |  |
| DSG4 | 18 | G | A | rs35378785 | G400R | 0.0044 | | 0.0043 | 0.0045 | 1.000 | | 0.487 | 5.33 | T | | D | 23.8 |  |
| BCR | 22 | C | A | rs12484731 | D752E | 0.024 | | 0.0155 | 0.0200 | 1.000 | | 0.672 | 3.98 | T | | B | 24.7 |  |
| BCR | 22 | A | G | rs35537221 | Y910C | 0.025 | | 0.0160 | 0.0207 | 1.000 | | 0.713 | 2.03 | T | | B | 23.7 |  |
| BCR | 22 | T | TCC  GG | rs372013175 | S1092SG? | - | | - | 1.228e-05 | - | | - | - | - | | - | - |  |
| TTN | 2 | A | G | rs62621206 | L22787P | 0.0056 | | 0.0103 | 0.0109 | 0.995 | | 0.461 | 4.66 | T | | P | 19.09 |  |
| TTN | 2 | T | C | rs72648982 | R7050G | 0.012 | | 0.0210 | 0.0221 | 0.165 | | 0.133 | 4.99 | T | | B | 13.38 |  |
| TTN | 2 | C | T | rs17355460 | G5624R | 0.0066 | | 0.0116 | 0.0118 | 1.000 | | 0.487 | 5.86 | T | | D | 22.5 |  |

Table 6. **Description of recessive compound heterozygous candidate variant identified with the ANNOVAR tool in the index case with ichthyosis.** **Gene**: Name of the gene. **Chr:** Chromosome. **Ref:** Reference allele. **Alt:** Alternate allele. **dbSNP:** dbSNP database variant Identifier. **Change:** Amino acid change. **1000G:** 1000 genomes database allele frequency. **ExAC:** Exome Aggregation Consortium database allele frequency. **gnomAD**: genome aggregation database allele frequency. **GenoCanyon:** Conservation scores with GenoCanyon tool (Conserved region=scores~1). **fitCons:** Conservation scores with fitCons tool: (Conserved region= ~1). **GERP++RS:** Conservation scores with GERP++RS tool (Conservation region=scores>4.4). **SIFT:** Pathogenicity prediction with SIFT tool: D=Deleterious, T=Tolerated), **Polyphen2HDIV:** Pathogenicity prediction with PolyPhem2 tool for Mendelian disease variants (D=Damaging, P=Possibly Damaging, B=Benign, U=Unknown). **CADD:** Pathogenicity scores with CADD tool (Deleterious=scores>14). *VCX3A: CTCGCTCTCCTGACTCAGTGGTTCCTCCACCTGGCTCTCCTGACTCAGTGGTTCCTCCAC/-.

**Clinical interpretation of recessive compound heterozygous candidate variant**

| **Gene** | **Chr** | **dbSNP** | **Change** | **Clinvar** | **Varsome** | **Intervar** | **ACMG** | **Effect** | **Severity** | **Pheno** |
| --- | --- | --- | --- | --- | --- | --- | --- | --- | --- | --- |
| VCX3A | X | rs74393938 | V186M | - | BS1, PB4 | BS1, PB4 | LB | Missense | Medium | 14.04 |
| VCX3A | X | - |  | - | - | - | VUS | Inframe indel | Medium | 14.04 |
| HRNR | 1 | rs773518265 | Q2469L | - | BP1, BP4 | BS1, BP4 | LB | Missense | Medium | 12.55 |
| HRNR | 1 | rs12116427 | T2270S | - | BS1, BP1, BP4 | BS1, BP4 | LB | Missense | Medium | 12.55 |
| HRNR | 1 | rs746911441 | GS2267 | - | - | - | VUS | Frameshift | High | 12.55 |
| HRNR | 1 | rs776354084 | Q2265QH? | - | - | - | VUS | Frameshift | High | 12.55 |
| HRNR | 1 | rs747830959 | D2264G | - | BS1, BP1, BP4 | BP4 | LB, VUS | Missense | Medium | 12.55 |
| HRNR | 1 | rs769619529 | F2263C | - | BS1, BP1, BP4 | BP4 | LB, VUS | Missense | Medium | 12.55 |
| HRNR | 1 | rs1266501645 | T2262S | - | BP1, BP4, PP2 | - | LB | Missense | Medium | 12.55 |
| HRNR | 1 | rs139799138 | R2167C | - | BS1, BP1, BP4 | BS1, BP4 | LB | Missense | Medium | 12.55 |
| HRNR | 1 | rs186602563 | S860T | - | BS1, BP1, BP4 | BS1 | LB, VUS | Missense | Medium | 12.55 |
| HRNR | 1 | rs76694305 | T616A | - | BP1, BP4 | BP4 | LB, VUS | Missense | Medium | 12.55 |
| HS6ST1 | 2 | rs3958533 | R249S | B, LB | BP6, PP3 | PM1 | VUS | Missense | High | 6.46 |
| HS6ST1 | 2 | rs199993343 | V114G | - | BP4 | BS1, PM1 | VUS | Missense | Medium | 6.46 |
| HS6ST1 | 2 | rs200979099 | D87E | LB | BP6, PP3 | PM1, PP3 | VUS | Missense | Medium | 6.46 |
| DSG4 | 18 | rs36040686 | S79L | B | BS1, BS2, BP4, BP6, PM1 | BS2, BP4, PM1 | B | Missense | Medium | 4.61 |
| DSG4 | 18 | rs35378785 | G400R | B | BS1, BS2, BP4, BP6 | BS1, BS2, PM1 | B | Missense | Medium | 4.61 |
| BCR | 22 | rs12484731 | D752E | - | BA1, BP1, BP4, BP6 | BS1, BS2, PM1 | B | Missense | Medium | 4.61 |
| BCR | 22 | rs35537221 | Y910C | - | BA1, BP1, BP4, BP6 | BS1, BS2, PM1 | B | Missense | Medium | 4.61 |
| BCR | 22 | rs372013175 | S1092SG? | - | PVS1, PP3 | - | VUS | Frameshift | High | 4.61 |
| TTN | 2 | rs62621206 | L22787P | CIOP | BS1, BS2, BP1, BP4, BP6, PM1, PP5 | PM2, PP2, PP5 | VUS | Missense | Medium | 2.06 |
| TTN | 2 | rs72648982 | R7050G | B, LB | BS1, BS2, BP1, BP4, BP6 | BS1, BP1, BP6, PM1, PP2 | B, LB | Missense | Medium | 2.06 |
| TTN | 2 | rs17355460 | G5624R | B | BS1, BS2, BP1, BP4, BP6 | BP1, BP6, PM1, PP2, PP3 | B, LB | Missense | Medium | 2.06 |

Table 7. **Clinical interpretation of the recessive compound heterozygous candidate variant identified in the exome analysis in the index case with ichthyosis**. **Gene**: Name of the gene. **Chr:** Chromosome. **dbSNP:** dbSNP database variant Identifier. **Change:** Amino acid change. **ClinVar:** Clinical significance in ClinVar. **Varsome:** Classification of variants according to the Varsome platform. **Intervar:** Classification of the variants according to the Intervar platform. **ACMG:** Classification of the variants according to the American College of Medical Genetics. **Effect:** The effect of the variant in the protein (including splicing effects). **Severity:** Severity of the impact on the protein based on the effect. **Pheno:** The score for the association between the gene and the phenotype terms (Ichthyosis) according to GTex platform. **B:** Benign, **LB:** Likely benign, **LP:** Likely pathogenic, **P:** Pathogenic, **VUS:** Variant with Uncertain Significance, **CIOP:** Conflicting interpretations of pathogenicity. **BA1:** Benign Stand-Alone, **BS1-BS4**: Benign Strong, **BP1-BP7**: Benign supporting, **PP1-PP5:** Pathogenic supporting, **PM1-PM6:** Pathogenic moderate, **PS1-PS6:** Pathogenic strong, **PVS1:** Pathogenic very strong.

Finally, we also looked for variants in heterozygous state, taking in count that *“de novo”* mutations could explain that the patient is the only individual affected within the family. We found 18 heterozygotic variants. All of them have a MAF minor 0.01 but only two variants (rs199727604 and rs185617318) are considered as deleterious by the pathogenicity predictors the others are considered as tolerated and benign variants by the pathogenicity predictors, Table 7. Nine variants are classified as benign or likely benign, eight as variant with uncertain significance and only one (rs185617318) as likely pathogenic according ACMG criteria. Three of the variants (rs199727604, rs200240573 and rs185617318) seem to have a high effect on protein, for the others the effect is considered as medium, Table 8.

**Dominant heterozygous candidate variant**

| **Genetic variant Information** | | | | | | **Allele Frequency** | | | | **Evolutionary conservation predictors** | | | | **Pathogenicity predictors** | | | |
| --- | --- | --- | --- | --- | --- | --- | --- | --- | --- | --- | --- | --- | --- | --- | --- | --- | --- |
| **Gene** | **Chr** | **Ref** | **Alt** | **dbSNP** | **Change** | **1000G** | **ExAC** | **gnomAD** | **Geno**  **Canyon** | | **fitCons** | **GERP++** | **SIFT** | | **Poly**  **Phen2** | **CADD** |  |
| KRT6B | 12 | G | A | rs199727604 | R443W | 0.0006 | 7.413e-05 | 6.091e-05 | 0.993 | | 0.526 | 0.502 | D | | D | 32 |  |
| TGM3 | 20 | A | C | rs201410609 | M492L | 0.0002 | 0.0001 | 0.0002 | 0.996 | | 0.497 | -10.3 | T | | B | 0.001 |  |
| COL7A1 | 3 | T | G | rs200240573 | - | 0.0002 | 8.246e-06 | 4.061e-06 | - | | - | - | - | | - | - |  |
| HRNR | 1 | T | C | rs747830959 | D2264G | - | 0.0021 | 0.0002 | 0.000 | | 0.554 | 0.55 | T | | B | 1.479 |  |
| HRNR | 1 | A | C | rs769619529 | F2263C | - | 0.0011 | 7.163e-05 | 1.000 | | 0.554 | -5.38 | T | | B | 0.001 |  |
| HRNR | 1 | G | C | rs1266501645 | T2262S | - | - | - | - | | - | - | - | | - | - |  |
| HRNR | 1 | T | C | rs76694305 | T616A | - | 1.647e-05 | 0.0039 | 0.032 | | 0.554 | -7.45 | T | | B | 0.002 |  |
| GP1BB | 22 | G | A | rs1033155055 | G106S | - | - | - | - | | - | - | - | | - | - |  |
| CARD14 | 17 | C | G | rs150536049 | S378R | 0.0004 | 0.0003 | 0.0004 | 0.163 | | 0.520 | 2.11 | T | | B | 10.12 |  |
| FLRT3 | 20 | C | G | - | G12A | - | - | - | - | | - | - | - | | - | - |  |
| DYSF | 2 | C | T | rs185617318 | R2044W | 0.0004 | 4.942e-05 | 6.497e-05 | 1.000 | | 0.707 | 4.17 | D | | D | 35 |  |
| CCPG1 | 15 | C | T | rs772623812 | A39T | - | 7.541e-05 | 5.299e-05 | 0.091 | | 0.732 | -5.75 | T | | B | 8.898 |  |
| CHIT1 | 1 | T | C | rs1362028418 | H64R | - | - | - | - | | - | - | - | | - | - |  |
| PCSK1 | 5 | C | T | rs1049269132 | E38K | - | - | - | - | | - | - | - | | - | - |  |
| ATN1 | 12 | ACAGCAGCAG | A,ACAGCAGCAGCAG | rs60216939 | Q491QQ | - | - | - | - | | - | - | - | | - | - |  |
| FANCM | 14 | A | G | rs199948045 | I818V | 0.0006 | 0.0002 | 0.0002 | 0.146 | | 0.615 | 0.668 | T | | B | 0.002 |  |
| FUT8 | 14 | C | T | rs181724726 | S66F | 0.0002 | - | 8.172e-06 | 0.999 | | 0.706 | 4.95 | T | | B | 21.9 |  |
| SLC22A5 | 5 | T | G | rs544332057 | S39A | 0.0002 | 9.315e-06 | 4.118e-05 | 1.000 | | 0.652 | -4.68 | T | | B | 0.005 |  |

Table 8. **Description of dominant heterozygous candidate variant identified with the ANNOVAR tool in the index case with ichthyosis.** **Gene**: Name of the gene. **Chr:** Chromosome. **Ref:** Reference allele. **Alt:** Alternate allele. **dbSNP:** dbSNP database variant Identifier. **Change:** Amino acid change. **1000G:** 1000 genomes database allele frequency. **ExAC:** Exome Aggregation Consortium database allele frequency. **gnomAD**: genome aggregation database allele frequency. **GenoCanyon:** Conservation scores with GenoCanyon tool (Conserved region=scores~1). **fitCons:** Conservation scores with fitCons tool: (Conserved region= ~1). **GERP++RS:** Conservation scores with GERP++RS tool (Conservation region=scores>4.4). **SIFT:** Pathogenicity prediction with SIFT tool: D=Deleterious, T=Tolerated), **Polyphen2HDIV:** Pathogenicity prediction with PolyPhem2 tool for Mendelian disease variants (D=Damaging, P=Possibly Damaging, B=Benign, U=Unknown). **CADD:** Pathogenicity scores with CADD tool (Deleterious=scores>14).

**Clinical interpretation of Dominant heterozygous candidate variant**

| **Gene** | **Chr** | **dbSNP** | **Change** | **Clinvar** | **Varsome** | **Intervar** | **ACMG** | **Effect** | **Severity** | **Pheno** |
| --- | --- | --- | --- | --- | --- | --- | --- | --- | --- | --- |
| KRT6B | 12 | rs199727604 | R443W | - | BS2, BP1, PM1, PP3 | BS2 | LB, VUS | Missense | High | 25.18 |
| TGM3 | 20 | rs201410609 | M492L | - | BP1,BP4, PM2 | BS2, BP1, BP4, PM1 | LB | Missense | Medium | 14.06 |
| COL7A1 | 3 | rs200240573 | - | - | BP2, PM2 | - | VUS | Intron, Splice site region | High | 13.75 |
| HRNR | 1 | rs747830959 | D2264G | - | BS1, BP1, BP4 | BP4 | LB, VUS | Missense | Medium | 12.55 |
| HRNR | 1 | rs769619529 | F2263C | - | BS1, BP1, BP4 | BP4 | LB, VUS | Missense | Medium | 12.55 |
| HRNR | 1 | rs1266501645 | T2262S | - | BP1, BP4, PP2 | - | LB | Missense | Medium | 12.55 |
| HRNR | 1 | rs76694305 | T616A | - | BP1, BP4 | BP4 | LB, VUS | Missense | Medium | 12.55 |
| GP1BB | 22 | rs1033155055 | G106S | - | BP2, PP2, PM1, PM2 | - | VUS | Missense | Medium | 7.72 |
| CARD14 | 17 | rs150536049 | S378R | LB | BS1, BS2, BP1, BP4, BP6 | BS2, PM2 | B, VUS | Missense | Medium | 6.46 |
| FLRT3 | 20 | - | G12A | - | - | - | VUS | Missense | Medium | 6.46 |
| DYSF | 2 | rs185617318 | R2044W | VUS | PM1, PM2, PP2, PP3 | PM1, PM2, PP3 | LP | Missense | High | 6.04 |
| CCPG1 | 15 | rs772623812 | A39T | - | BS1, BP1, BP4 | BP4, PM2 | LB, VUS | Missense | Medium | 4.61 |
| CHIT1 | 1 | rs1362028418 | H64R | - | BP4, PM1, PM2 | - | VUS | Missense | Medium | 3.57 |
| PCSK1 | 5 | rs1049269132 | E38K | - | BP4, PP2, PM2 | - | VUS | Missense | Medium | 2.26 |
| ATN1 | 12 | rs60216939 | Q491QQ | - | - | - | VUS | Inframe indel | Medium | 2.06 |
| FANCM | 14 | rs199948045 | I818V | VUS | BP4, PM2 | BP4 | LB | Missense | Medium | 2.06 |
| FUT8 | 14 | rs181724726 | S66F | - | BP4, PM2 | - | VUS | Missense | Medium | 0.62 |
| SLC22A5 | 5 | rs544332057 | S39A | - | BP4, PP2, PM1, PM2 | BP4, PM2 | VUS | Missense | Medium | 0.2 |

Table 9. **Clinical interpretation of the dominant heterozygous candidate variant identified in the exome analysis in the index case with ichthyosis**. **Gene**: Name of the gene. **Chr:** Chromosome. **dbSNP:** dbSNP database variant Identifier. **Change:** Amino acid change. **ClinVar:** Clinical significance in ClinVar. **Varsome:** Classification of variants according to the Varsome platform. **Intervar:** Classification of the variants according to the Intervar platform. **ACMG:** Classification of the variants according to the American College of Medical Genetics. **Effect:** The effect of the variant in the protein (including splicing effects). **Severity:** Severity of the impact on the protein based on the effect. **Pheno:** The score for the association between the gene and the phenotype terms (Ichthyosis) according to GTex platform. Database. **BA1:** Benign Stand-Alone, **BS1-BS4**: Benign Strong, **BP1-BP7**: Benign supporting, **PP1-PP5:** Pathogenic supporting, **PM1-PM6:** Pathogenic moderate, **PS1-PS6:** Pathogenic strong, **PVS1:** Pathogenic very strong.

**References**

1. Chen R, Im H, Snyder M. Whole-exome enrichment with the agilent sureselect human all exon platform. Cold Spring Harb Protoc . 2015 Jul 1;2015(7):626–33. Available from: https://pubmed.ncbi.nlm.nih.gov/25762417/

2. Babraham Bioinformatics - FastQC A Quality Control tool for High Throughput Sequence Data. Available from: https://www.bioinformatics.babraham.ac.uk/projects/fastqc/

3. the Burrows-Wheeler Aligner tool, bwa-0.7.12. Available from: http://bio-bwa.sourceforge.net/bwa.shtml

4. Marthey S. Genome Analysis Toolkit. 2013;

5. Pablo Cingolani. SnpEff tool. Available from: http://snpeff.sourceforge.net/SnpEff.html

6. Chang X, Wang K. Wannovar: Annotating genetic variants for personal genomes via the web. J Med Genet. 2012 Jul;49(7):433–6. Available from: https://pubmed.ncbi.nlm.nih.gov/22717648/

7. Dahary D, Golan Y, Mazor Y, Zelig O, Barshir R, Twik M, et al. Genome analysis and knowledge-driven variant interpretation with TGex. BMC Med Genomics. 2019 Dec 30;12(1). Available from: https://pubmed.ncbi.nlm.nih.gov/31888639/

8. Kopanos C, Tsiolkas V, Kouris A, Chapple CE, Albarca Aguilera M, Meyer R, et al. VarSome: the human genomic variant search engine. Bioinformatics. 2019 Jun 1;35(11):1978–80. Available from: https://pubmed.ncbi.nlm.nih.gov/30376034/

9. Li Q, Wang K. InterVar: Clinical Interpretation of Genetic Variants by the 2015 ACMG-AMP Guidelines. Am J Hum Genet. 2017 Feb 2;100(2):267–80. Available from: https://pubmed.ncbi.nlm.nih.gov/28132688/

10. Richards S, Aziz N, Bale S, Bick D, Das S, Gastier-Foster J, et al. Standards and guidelines for the interpretation of sequence variants: A joint consensus recommendation of the American College of Medical Genetics and Genomics and the Association for Molecular Pathology. Genet Med. 2015 May 8; 17(5):405–24. Available from: https://pubmed.ncbi.nlm.nih.gov/25741868/
